# Percutaneous Mitral Valve Interventions (Repair): Current Indications and Future Perspectives

**DOI:** 10.3389/fcvm.2019.00088

**Published:** 2019-07-12

**Authors:** Mahek Shah, Ulrich P. Jorde

**Affiliations:** Department of Cardiology, Montefiore Medical Center, Bronx, NY, United States

**Keywords:** mitral regurgitation, functional mitral regurgitation, percutaneous mitral repair, MitraClip device, degenerative mitral regurgitation (DMR), heart failure, medical management, mitral surgery

## Abstract

Mitral valve regurgitation (MR) is the commonest valvular abnormality encountered among adult patients with cardiac valvular disease and conveys significant morbidity and mortality. The mitral valve is a complex anatomical structure and etiology for regurgitation is classified as either *primary* or *secondary* MR. Identification of the etiology in severe MR is critical in determining the appropriate treatment strategy. Transcatheter mitral valve repair (TMVR) is a minimally invasive technique for treatment of selected patients with symptomatic chronic moderate-severe or severe (3 to 4+) MR. While surgery remains the mainstay for treatment in *primary* MR, several technological advances within the last decade have made transcatheter mitral valve intervention increasingly feasible and safe in clinical practice. Use of TMVR in patients with severe MR has successfully reduced patient symptoms, disease morbidity, improved quality of life, and facilitated reverse remodeling with potential for a survival advantage among certain patients with *secondary* MR. Recent randomized controlled trials on MitraClip use in *secondary* MR have reinvigorated interest in this disease and refocused our attention on optimizing patient selection and timing of intervention to maximize benefit from using such percutaneous devices. In our review, we discuss etiologies and pathophysiology in both acute MR and development of chronic severe MR. We discuss management strategies for MR among patients based on etiology, particularly percutaneous mitral valve interventional therapies. We perform an extensive review comparing and contrasting existing data on safety, efficacy, durability, and appropriate patient selection related to MitraClip implantation in both *primary* and *secondary* MR. Lastly, we explore percutaneous MV therapies beyond the MitraClip as we await larger scale trials on these devices prior to them making way into day-to-day practice.

## Structure and Anatomy of the Mitral Valve

**Figure 1:**
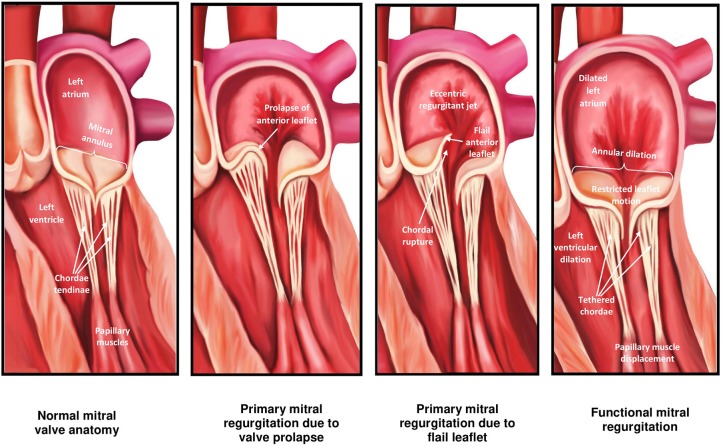
Mitral valve apparatus and etiologies for mitral regurgitation.

**Figure 2:**
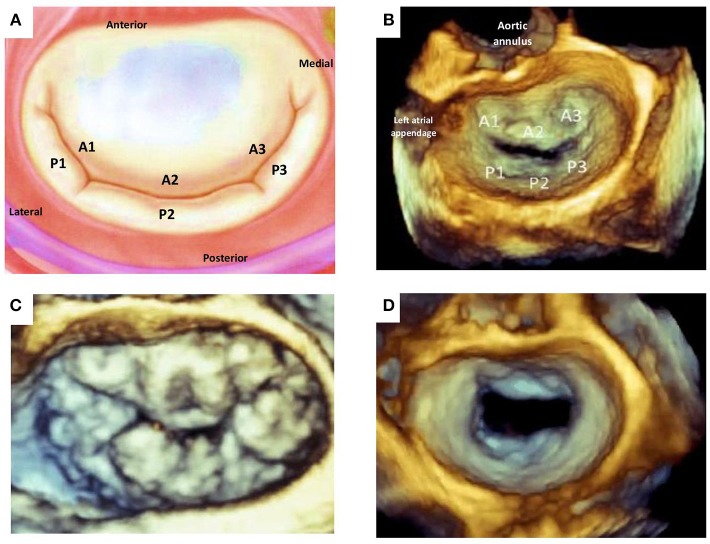
Mitral valve leaflet anatomy. **(A)** Schematic of normal mitral valve. **(B)** Corresponding 3D TEE view of the atrial aspect of normal mitral valvular anatomy. **(C)** 3D TEE image of a patients with multi-leaflet prolapse (Barlow's disease). **(D)** 3D TEE image of incomplete central closure of mitral valve during systole and resultant severe functional mitral regurgitation. TEE, transesophageal echocardiography.

The mitral valve (MV) is complex and involves synchronous participation of several anatomical structures including valvular leaflets, chordae tendinae, papillary muscles, mitral annulus, and left ventricular (LV) myocardium to facilitate unidirectional passage of blood from left atrium into the ventricle during diastole, and preventing regurgitation during systole (1) (Figure 1). Anatomical changes at any level can potentiate valvular dysfunction particularly abnormal leaflet closure and regurgitation of blood. The MV comprises of anterior (aortic) and posterior (mural) leaflets, with three segments each (A1A2A3, P1P2P3) labeled from the lateral to medial aspect of heart (Figure 2). The posterior leaflet has more prominent notching along its free edge, clearly dividing the leaflet into three scallops or segments. The mitral annulus is a saddle shaped structure composed of fibrocollagenous tissue attached to the mitral leaflets. The anterior portion of the mitral annulus is attached to the fibrous trigones which contain conduction tissue whereas the posterior annulus is less well-developed, more muscular and prone to dilation. Mitral valvular regurgitation is the commonest valvular disorder among adults (2, 3). Failure of complete coaptation and adequate symmetrical apposition of both mitral leaflets results in varying degree of MR. Identifying the etiology for failure of underlying MV function can aid in developing an appropriate treatment strategy.

## Etiology of MR

**Figure 3:**
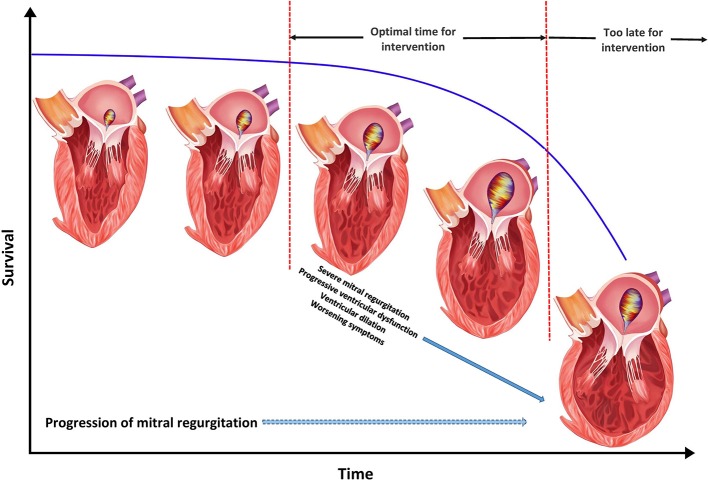
Optimal timing for mitral valve intervention in functional mitral regurgitation (MR). The figure represents the natural history of functional MR with progression in severity of MR over time accompanied by left ventricular dysfunction, ventricular dilation, progressive symptoms, and worsening survival.

When it comes to understanding the etiology of MR, designating the MR as either “*primary* or *degenerative*” (related to anatomical abnormalities in valve leaflets and/or chordae tendinae) vs. “*secondary* or *functional*” (usually related to systolic tethering of anatomically intact MV leaflets due to annular dilation in the setting global or regional LV wall motion abnormalities) is commonly the initial step [Table 1; Figures 1, 2; (3)]. In *functional* MR, the LV becomes more spherical and this is associated with retraction of the papillary muscles and chordae tendinae along with widening separation of the valvular leaflets. In most cases, MR worsens over time and has a relatively chronic picture. Less commonly presentation can be acute when severe MR results from either rupture of chordae tendinae or papillary muscle and infective endocarditis. In the developed world, the commonest etiology for MR is likely *degenerative* MV disease as a result of the high prevalence of MV prolapse (MVP) in the general population from myxomatous degeneration and chordal stretching (4). However, in one single-center study evaluating 1,095 patients with significant MR and heart failure (HF) symptoms, *functional* MR (~75%) was more common followed by *degenerative* MR (5). An additional etiology for mitral regurgitation has been noted among patients with isolated atrial fibrillation in the presence of normal mitral leaflet, subvalvular and LV anatomy called “*atrial functional*” MR. It has been attributed to left atrial enlargement and dilation in mitral annulus as the primary mechanism for mitral leaflet malcoaptation (6). Such a new classification for MR solely secondary to dilation of the mitral annulus has been debated and the prevalence of *atrial functional* MR in prior MR studies is somewhat unknown due to its poor recognition as a separate entity (7). While both classes of atrial and ventricular *functional* MR have been associated with normal leaflet anatomy, accumulating data seems to suggest that alterations in the extracellular matrix within the mitral leaflets and insufficient leaflet remodeling relative to the increase in mitral annulus also contribute to worsening of MR (8–10).

**Table 1:** Characteristics based on etiology of mitral regurgitation.

	**Primary MR**	**Secondary MR**
Prevalence	Higher mainly due to MV prolapse	Lower in general population
Mechanism	Pathology of ≥1 of the components of the valve (leaflets, chordae tendinae, papillary muscles, annulus)	Left ventricular dysfunction with papillary muscle displacement, LV dyssynchrony, associated leaflet tethering and annular dilation. Normal (or nearly normal) mitral leaflet and chordal structure
Associated diseases	• Myxomatous valve - Barlow's disease, Fibroelastic deficiency disease • Rheumatic valvular disease • Endocarditis • Radiation therapy, connective tissues disease, drug induced, mitral annular calcification, cleft mitral valve	• Dilated cardiomyopathy • Ischemic MR secondary to previous myocardial infarction • Hypertrophic cardiomyopathy
Carpentier functional classification type^*^	• Type I (leaflet perforation or cleft) • Type II (MV prolapse) • Type IIIa (rheumatic valve disease, drug induced MR, mitral annular calcification)	• Type I (atrial MR, non-ischemic cardiomyopathy) • Type IIIb (ischemic cardiomyopathy, LV dysfunction and systolic leaflet tethering)

* *Type 1: normal leaflet motion. Type 2: excessive leaflet motion. Type 3a: leaflet restriction in systole and diastole. Type 3b: leaflet restriction in systole*.

## Pathophysiology of Acute and Chronic MR

### Acute MR

Acute MR results in acute left atrial and LV volume overload, increasing ventricular preload and stroke volume as consequence of the Frank-Starling mechanism. In addition, there is a reduction in LV systolic wall stress and afterload with increase in LV ejection fraction (EF). The acute increase in volume from MR into a non-compliant left atrium results in marked elevation in left atrial and pulmonary venous pressures, causing pulmonary edema.

### Chronic MR

As patients evolve from acute to chronic MR, the LV dilates and changes from a small hyperkinetic chamber in acute MR to a large compliant chamber (11). During this transition, rearrangement of myocardial fibers and addition of sarcomeres results in eccentric LV hypertrophy (12). In the early compensated phase of chronic MR, the LV is able to maintain normal wall stress, high stroke volume and adequate cardiac output at the expense of increased LV end diastolic volume (LVEDV). These temporal changes in LV structure result in normalized preload and afterload at the sarcomere level and thus compensated chronic MR. During this phase, the left atrium enlarges in size with improvement in atrial compliance and decline in pulmonary venous pressures. As the underlying disease progresses, however, usually over years the LV dilates further, afterload increases and LV contractility eventually worsens with decompensation of disease status (13). The underlying pathophysiology for *atrial functional* MR is less well-studied, and likely related to left atrial enlargement, displacement of posterior annulus onto the crest of the LV, close apposition of posterior mitral leaflet to the LV wall, reduction in posterior leaflet area for coaptation, and counterclockwise torque of the anterior mitral annulus causing tethering of the anterior mitral leaflet with leaflet tenting (14). While patients are often asymptomatic during the compensated stage of disease, there is growing interest in timing intervention for MR early to prevent decompensation. Recent trials on percutaneous MV repair have rejuvenated interest on the interplay between LV dysfunction and degree of MR, to identify a phenotype more responsive to intervention.

## Disease Prognosis and Natural History

Severe untreated MR has a fairly poor prognosis irrespective of etiology. In addition to reduced survival, several data point to worse quality of life and a time dependent increase in the burden of atrial fibrillation and HF symptoms with severe MR. Factors associated with worse outcomes among patients with severe MR can be seen in Table 2 (15–19). Evolution of MR into the chronic compensated and decompensated stages occurs over many years to decades, depending on severity of the MR and cardiac structural changes. The 2014 American Heart Association/American College of Cardiology (AHA/ACC) Guideline for the Management of Patients With Valvular Heart Disease and 2017 focused update describe the nature of this transition to more advanced disease by defining stages for clinical evaluation combining patient's functional status and hemodynamic data as seen in Table 3 (3, 20).

**Table 2:** Factors associated with worse outcomes with significant MR.

**Factors associated with worse outcomes with significant MR**
• Development of heart failure symptoms (Survival worse in NYHA functional class III/IV) • New atrial fibrillation • Right ventricular dysfunction^*^ • Severe tricuspid regurgitation^*^ • Functional etiology • Echocardiographic parameters - Effective regurgitant orifice area ≥40 mm^2^ (primary MR) - Effective regurgitant orifice area ≥20 mm^2^ (secondary MR) - LV ejection fraction <60% (LV systolic dysfunction)

* *When studied with functional mitral regurgitation*.

**Table 3:** Stages of mitral regurgitation in chronic primary and secondary MR.

**Grade**	**Definition**	**Valve hemodynamics**	**Symptoms**
A	At risk for MR	• No jet or small central jet area < 20% LA • VC < 0.3 cm	None
B	Progressive MR	• Central jet MR 20–40% LA or late systolic eccentric jet MR • VC < 0.7 cm • Rvol < 60 ml • RF < 50% • ERO < 0.4 cm^2^ • Angiographic grade 1 to 2+	None
C	Asymptomatic severe MR	• Central jet MR >40% LA or holosystolic eccentric jet MR • VC ≥0.7 cm • Rvol ≥60 ml • RF ≥50% • ERO ≥0.4 cm^2^ • Angiographic grade 3 to 4+	None
D	Symptomatic severe MR	• Central jet MR >40% LA or holosystolic eccentric jet MR • VC ≥0.7 cm • Rvol ≥60 ml • RF ≥50% • ERO ≥0.4 cm^2^ • Angiographic grade 3 to 4+	Decreased exercise tolerance Exertional dyspnea

*MR, mitral regurgitation; VC, vena contracta; Rvol, regurgitant volume; RF, regurgitant fraction; ERO, effective orifice area; LA, left atrium*.

The compensated phase of MR is considered benign without overt dilation of LV [LV end diastolic diameter (LVEDD) <60 mm, end systolic diameter (LVESD) <40 mm, end diastolic volume (LVEDV) <110 ml/m^2^, end systolic volume <45 ml/m^2^ and ejection fraction >60%], low arrhythmia burden and being relatively asymptomatic with mild to moderate exertion. The decompensated phase of disease is based upon presence of HF symptoms and suboptimal LV parameters secondary to failure of compensatory mechanisms (LVEDD >70 mm, LVESD >47 mm, LVEDV >160 ml/m^2^, LVEDV >60 ml/m^2^, LV ejection fraction <50%). The transition phase between these two disease phenotypes is less well-defined with structural changes in the intermediate range and variable symptom severity but finds itself as the central focus for ideal timing of MV intervention to halt progression of MR and LV remodeling (Figure 3) (21–24).

## Patient Selection for Intervention in Chronic MR

To understand patient selection, we ought to better understand staging of MR and its relation to MR severity. Stages A and B represent mild-moderate forms of disease where periodic monitoring is recommended, whereas stages C and D represent asymptomatic and symptomatic severe MR, respectively. Further classification of stage C depends upon LV function and size (C1- LVEF >60% and LVESD ≤ 40 mm; C2- LVEF ≤ 60%; and LVESD >40 mm). Chronic severe (*primary* or *secondary*) MR is identified by the presence of a combination of the following echocardiographic criteria: central jet of MR >40% of left atrium or holosystolic eccentric MR, vena contracta ≥0.7 cm, regurgitant volume ≥60 mL, regurgitant fraction ≥50% and an effective regurgitant orifice area (EROA) ≥0.40 cm^2^ (20). The corresponding angiographic grade for severe MR is 3 to 4+.

The only effective therapy for severe *primary* MR is valve repair or valve replacement. Based on the 2017 update to 2014 AHA/ACC valvular guidelines, decision regarding candidacy for intervention in chronic *primary* MR is dependent on disease severity, symptom status, LV size and function, rest or exercise pulmonary hypertension, new onset atrial fibrillation, likelihood for successful repair and patient preference. Intervention for severe chronic *functional* MR is less well-studied as can be observed by the lack of a strong recommendation for mitral valve surgery among these guidelines. Guidelines are yet to be updated to reflect recent data on use of percutaneous MV therapies such as the MitraClip in functional MR, considering the potential for improvement in patient level outcomes among selected individuals with severe *functional* MR.

## Management of Acute MR

While prompt surgery is recommended in all patients with acute severe symptomatic MR, vasodilator therapies and percutaneous devices such as intra-aortic balloon pump or Impella can be used in the interim to stabilize patients in preparation for surgery (20). Valve repair is preferable over valve replacement in acute management of these patients, however the ability to repair MV is often limited by more extensive disease involving the MV apparatus (25). The role of percutaneous repair in acute MR will be discussed in the section on percutaneous therapies below.

## Management of Chronic MR

### Primary MR

Medical therapy has a limited to no role in the treatment of *primary* MR, however, appropriate guideline directed medical therapy is recommended in patients with hypertension or HF with reduced ejection fraction. Surgical therapy is the treatment of choice in treatment of *primary* MR. Intervention once MR is already in the decompensated phase is accompanied with high morbidity and mortality due to recurrence of HF (26). Progression of MR severity as noted by reduction in EF to <60% or LV dilation to LVESD >40 mm is a high risk marker prior to surgery and intervention should take place before such changes occur in chronic forms of MR. The decision to intervene is complicated by the fact that some of the asymptomatic patients remain asymptomatic and stable for years whereas others develop irreversible LV systolic dysfunction. In one recent paper, the authors retrospectively followed 82 asymptomatic patients with MVP, normal ejection fraction and mild to moderate MR for a mean of 4.5 years. They found that none of the patients with mild MR progressed to severe MR, whereas 50% with moderate MR progressed to severe MR. No clinical variables or echocardiographic parameters predicted progression of disease apart from mitral annular diameter of 39.6 mm (sensitivity 100%, and specificity 63.8%)(27). The role for clinical variables such as male sex, older age, atrial fibrillation, higher weight and hypertension or echocardiographic parameters such as valvular thickening in predicting progression of disease is controversial (27–29). In summary, there are no clear predictors to which patients tend to progress, making serial clinical and echocardiographic monitoring standard of care while on medical therapy.

MV repair is the preferred mode of therapy considering the lower operative mortality, superior long-term survival, and fewer valve related complications from bleeding and endocarditis compared to valve replacement (30, 31). Early repair has been shown to approximate outcomes in age-matched controls, extending potential benefit to asymptomatic or minimally symptomatic patients with MV disease feasible for repair at low operative risk.

### Secondary MR

Pharmacologic therapy comprising of a combination of angiotensin receptor neprilysin inhibitors, angiotensin converting enzyme inhibitors, angiotensin receptor blockers, beta blockers, mineralocorticoid receptor antagonists, and diuretics is recommended in the management of HF with reduced ejection fraction and severe MR (32). Use of cardiac resynchronization therapy among selected patients with LV dysfunction and dyssynchrony manifested by widening of the QRS complex on electrocardiogram is known to improve *secondary MR*. Cardiac resynchronization therapy produces marked reductions in LVESD, LVEDD and MR severity amongst responders (33, 34). Treatment of *secondary MR* includes addressing concurrent conditions such as atherosclerotic coronary artery disease in the presence of LV dysfunction via percutaneous or surgical revascularization. According to the recent valvular guidelines, a weak recommendation (Level of recommendation: Class IIb) exists for surgical intervention in patients with severely symptomatic grade 3 to 4+ *secondary MR* despite optimum guideline-directed management, treatment of coronary disease and cardiac resynchronization therapy (35). In general, neither MV replacement nor repair has been shown to improve survival in the treatment of severe *functional* MR, only symptoms. In recent randomized controlled trials of moderate or severe ischemic MR and mildly reduced ejection fraction, mitral valve repair, or chordal-sparing mitral valve replacement failed to achieve long-term favorable effects on clinical outcomes while failing to show compelling evidence for LV reverse remodeling (36, 37). At 2 years, subgroup analysis did demonstrate favorable reverse remodeling that was most evident among patients undergoing repair but had no recurrence in MR (38). On the other hand, MitraClip placement in a specific group of patients with disproportionately severe *functional* MR was shown to improve outcomes including survival as described in section below. Management of *atrial functional* MR remains understudied and its primary mechanism is related to atrial remodeling due to atrial fibrillation. Measures directed toward halting or reversing atrial enlargement in atrial fibrillation such as rhythm control or ablation strategies may be beneficial but their superiority to rate control in reversing atrial remodeling has not been studied.

### Percutaneous MV Repair

Percutaneous MV repair is a minimally invasive approach to treatment of certain patients with symptomatic chronic significant MR. A multidisciplinary heart team (including general cardiologists, interventional cardiologists, cardiac surgeons, imaging specialists, HF specialists, and cardiac anesthesiologists) is recommended to evaluate and direct care among potential candidates for percutaneous valve repair. Currently, the only US Food and Drug Administration (FDA) approved device for percutaneous MV repair in *primary* and *secondary* MR is the MitraClip. Transcatheter MV repair is one of the fastest growing fields in structural heart disease intervention with constantly evolving safety and efficacy data on multiple novel device systems.

#### MitraClip

**Figure 4:**
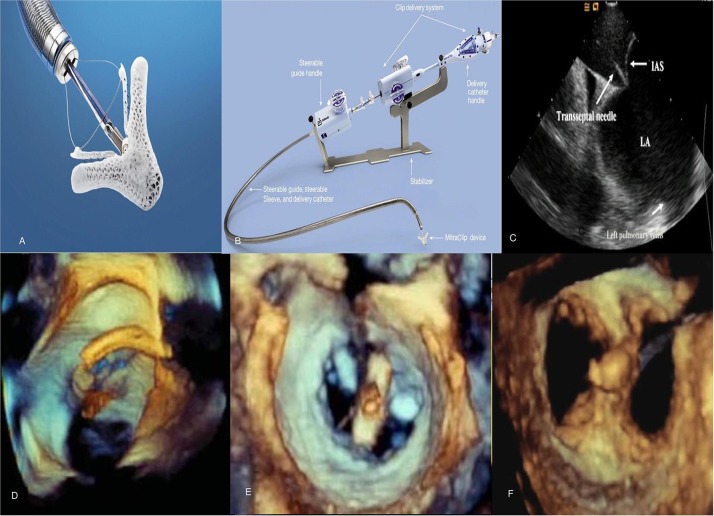
Mitraclip system and echocardiographic images during the procedure. **(A)** MitraClip device has 2 arms and 2 grippers fabricated with metal alloys and polyester fabric. **(B)** The steerable guide catheter and clip delivery system. **(C)** Transseptal puncture using intracardiac echocardiography to enter left atrium. **(D,E)** Stepwise positioning of the MitraClip perpendicular to axis of mitral valve adjacent to the A2-P2 scallops as seen on 3D TEE. **(F)** Post-MitraClip deployment double-orifice mitral valve seen on 3D TEE. TEE, transesophageal echocardiography.

The MitraClip (Abbott Laboratories, Menlo Park, California, USA) is a cobalt chromium clip covered with a polypropylene fabric, has two arms and works by grasping and approximating edges of the anterior and posterior valvular leaflet segments (Figure 4) in patients with severe MR. It is a catheter-based technology that was designed after the surgical Alfieri technique which connects the middle segment of the anterior leaflet to the middle scallop of the posterior leaflet of a regurgitant MV (39). MitraClip received initial CE-Mark approval in Europe in 2008 and was approved by the FDA in 2013 for use in *primary* MR and 2019 for use in *functional* MR.

#### Procedure Technique

The percutaneous procedure is performed with the patient under general anesthesia using transthoracic, transesophageal echocardiography and fluoroscopic guidance in the cardiac catheterization laboratory. The MitraClip procedure consists of several steps following femoral venous access [(40); Figure 4]:

1. Transseptal puncture—In *primary* MR, the puncture site needs to be roughly 5 cm above the mitral annulus to allow sufficient catheter and clip maneuvering. In *functional* MR the puncture site needs to be more inferior and closer to the annular plane (about 3.5 cm above annular plane) since tethering of leaflets results in coaptation occurring below the plane of the mitral annulus.
2. Advancement of guide catheter and delivery system into the left atrium—A stiff guidewire is passed into the left atrium and the trans-septal apparatus is exchanged for the guide catheter. The clip delivery system is then introduced into the guide catheter and the clip is advanced into the left atrial chamber.
3. Positioning of the MitraClip into the left ventricle to just below the MV leaflets—The clip delivery system is steered until it is aligned over the origin of the regurgitant jet, its arms opened to orient it perpendicular to MV coaptation and advanced into the ventricle just below the leaflet edges.
4. Grasping the leaflet edges, confirming position and releasing the clip—The clip is closed to 120° and pulled back until the mitral leaflets are captured in the arms of the clip. The clip is incrementally closed, while its position, leaflet attachment and the degree of MR can be assessed. Prior to the final release, the clip can be reopened and repositioned if needed. After adequate reduction of MR is ensured, the clip is released from the delivery system and all catheters are withdrawn. In cases with residual MR, additional clips can similarly be placed in the way of regurgitant jets while ensuring no evidence of significant *de novo* mitral stenosis. The entire procedure is performed on intravenous heparin while serially checking activated coagulation time (goal >250 s). After the clip is placed, patients are treated with aspirin 325 mg daily for 6–12 months and clopidogrel 75 mg daily for 30 days. These recommendations are based on estimated time to device endothelialization.

### MV Suitability

To facilitate safe positioning of the clip, pre-procedural evaluation of certain mitral valvular anatomical criteria (EVEREST criteria) has been recommended previously to identify eligibility. Planimetered MV area ≥4.0 cm^2^, minimal leaflet calcification in the grasping area, coaptation length of >2 mm, coaptation depth of <11 mm and in the case of degenerative disease, a flail gap of <10 mm and a flail width of <15 mm are considered favorable characteristics for MitraClip placement.

Despite the relatively stringent criteria described above, previous studies have demonstrated high rates of device success after MitraClip among patients with more complex MV anatomy including larger LV dimensions, severely reduced LV function and patients not meeting criteria for coaptation depth, coaptation length, and flail gap (41, 42). Durability of repair has been confirmed on intermediate term follow-up (1–3.5 years) depending on the study, however, a greater risk for re-intervention exists when implantation is performed beyond the above mentioned EVEREST criteria (42). In mid-2018, the US FDA approved the third generation of the MitraClip system with advanced steering, navigational and positioning clip capabilities to improve deliverability and precision of device. The new MitraClip NT_R_ device offers the original clip size with improved delivery system and the MitraClip XT_R_ device offers 3 mm longer clip arms and expands grasping reach of the device by 5 mm compared to the NT_R_ device (**Figure 6**). These developments have made more anatomically challenging valves favorable to successful edge-to-edge repair using the newer generation MitraClip devices.

Among contraindications to MitraClip placement are inability to tolerate procedural anticoagulation or antiplatelet agents post-procedure, active MV endocarditis, rheumatic MV disease, mitral stenosis from any cause and thrombosis of femoral access vein, inferior vena cava or left sided intracardiac structures.

### Complications of MitraClip

The risks for complications is low following MitraClip placement with rates comparable to open repair and the procedure being quite well-tolerated among recipients. Complications include access site bleeding, clip detachment from a single leaflet, device embolization, and development of mitral stenosis. In the first large scale trial evaluating MitraClip use i.e., the EVEREST II clinical trial, major adverse events of death and major stroke were similar patients receiving MitraClip and those undergoing MV surgery (43). On one hand, patients undergoing surgery needed more blood transfusions and longer mechanical ventilation whereas MitraClip implantation was associated with greater onset of new atrial fibrillation and acute renal failure. Rate of 30-day complications is usually in the range of 15–19% following such transcatheter MV repair (43, 44). Bleeding is largely peri-procedural from the vascular access site for MitraClip due to its large sheath size. Partial clip detachment is most common in the first year post-procedure but occurs in <5% cases. Clip embolization and complete detachment or hemodynamically significant mitral stenosis are rare. Risk for endocarditis involving the MitraClip is unclear since most data comes from case reports and use of peri-procedural antibiotic prophylaxis among recipients of MitraClip is controversial (45).

### Clinical Application

#### Chronic Primary MR

The 2017 focused update of the 2014 AHA/ACC valve guidelines suggested use of MitraClip in chronic severe *primary* MR (3 to 4+) among those who were highly symptomatic (New York Heart Association class III to IV) despite optimal guideline-directed medical therapy (stage D), had favorable anatomy, reasonable life expectancy and a prohibitive surgical risk due to comorbidities (Table 4). For patients with *primary* MR who met all criteria, the next step involves referral to the Heart Team for feasibility and potential risk vs. benefit from procedure. Most of these recommendations were made mainly in light of data from the EVEREST II trial.

**Table 4:** Factors that determine prohibitive surgical risk among patients with primary MR undergoing MitraClip evaluation.

**Prohibitive surgical risks to mitral valve surgery**
30-day Society of Thoracic Surgeons (STS) predicted operative mortality risk score of ≥8%
Porcelain or highly calcified aorta
Patient frailty
Severe liver disease
Severe pulmonary hypertension
Right ventricular dysfunction with severe tricuspid regurgitation
Others- chemotherapy for malignancy, major bleeding diathesis, immobility, AIDS, severe dementia

In the EVEREST II trial, 279 patients with 3+ to 4+ MR were randomized in 2:1 fashion to undergo either percutaneous repair, i.e., MitraClip device (184 patients) or mitral-valve surgery (95 patients) (43). Among patients undergoing MV surgery, 86% underwent MV repair and the rest underwent replacement. At least three of the following echocardiographic criteria were used to define moderate-severe (3+) or severe (4+) MR: (i) regurgitant color flow jet that was central and large (>6 cm^2^ or >30 percent of left atrial area) or smaller if eccentric, encircling the left atrium, (ii) pulmonary vein flow showing systolic blunting or systolic flow reversal, (iii) vena contracta ≥0.5 cm in the parasternal long axis view, (iv) regurgitant volume ≥45 ml/beat, (v) regurgitant fraction ≥40 percent, and (vi) regurgitant EROA ≥0.30 cm^2^. All patients were required to have a primary regurgitant jet from malcoaptation of the middle segments of the anterior and posterior leaflets at recruitment. ~3/4ths of the patients who made it into the study had *degenerative* MR.

The primary composite end point for efficacy was freedom from death, from surgery for MV dysfunction and from ≥3 grade residual MR. At 12-months, the end point was more frequent in the surgery group (73 vs. 55%) due to the higher rate of subsequent surgery for MV dysfunction in the MitraClip arm (20 vs. 2%). Mortality and ≥3 grade residual MR were similar in the two groups at ≈6 and ≈20%, respectively. Major complications at 30-days were higher in the surgical arm largely from a higher rate of transfusing ≥2 units of blood among patients undergoing surgery. More recently published 5-year data shows that patients who received MitraClip continued to have higher rates of repeat surgery and residual MR compared to the surgical arm, without any difference in overall mortality [(46); Table 5]. These data are reassuring for safety of MitraClip implantation in *primary* MR, but highlights the need for appropriate patient selection since more than a quarter of the patients needed repeat surgery at 5-years post-MitraClip placement. The majority of repeat surgery was still performed during the first year of follow up, lending credibility to the durability of a successful MV repair using the MitraClip.

**Table 5:** MitraClip trials on treating patients with severe mitral regurgitation.

**Study**	**Design**	**Comparison groups**	**Etiology for MR**	**Study endpoints**
Feldman et al. EVEREST II Trial 5-Year Results (46)	Prospective, multi-center, randomized controlled trial	2:1 MitraClip (*n* = 178) vs. MV surgery (*n* = 80)	73% primary MR, 27% functional MR	44.2 vs. 64.3% (*p* = 0.01)^*^ 12.3 vs. 1.8% (*p* = 0.02)^§^ 27.9 vs. 8.9% (*p* = 0.003)^€^ 20.8 and 26.8% (*p* = 0.4)^¥^
Stone et al. COAPT Trial (47)	Prospective, multi-center, randomized controlled trial	1:1 MitraClip (*n* = 302) vs. medical therapy (*n* = 312)	100% functional MR with LV dysfunction	35.8 vs. 67.9% (*p* < 0.001)^**^ 5.2 vs. 53.1% (*p* < 0.001)^§§^ 29.1 vs. 46.1% (*p* < 0.001)^¥¥^
Obadia et al. MITRA-FR Trial (48)	Prospective, open label, multi-center, randomized controlled trial	1:1 MitraClip (*n* = 152) vs. medical therapy (*n* = 152)	100% functional MR with LV dysfunction	54.6 vs. 51.3% (*p* = 0.53)^***^ 48.7 vs. 47.4% (*p* > 0.05)^§§§^ 24.3 vs. 22.4% (*p* > 0.05)^¥¥¥^

* *Composite endpoint: freedom from death, surgery, or 3+ or 4+ MR according to as treated analysis*.

§ *Rates of residual ≥3+ MR*.

€ *Rate of repeat surgery*.

¥ *Five-year mortality rates according to as treated analysis*.

** *Heart failure hospitalization within 24 months*.

§§ *Rate of residual ≥3+ MR at 12 months*.

¥¥ *Death from any cause at 24 months*.

*** *Composite primary outcome: death from any cause or unplanned hospitalization for heart failure at 12 months*.

§§§ *Unplanned heart failure hospitalization at 12 months*.

¥¥¥ *Death from any cause at 12 months*.

The quality of life benefits seen with MitraClip early on persisted on subsequent follow-up of the patients enrolled in EVEREST II with the proportion of patients with NYHA class III/IV symptoms decreasing to 5.7% at 4 years from 45% at baseline. Such improvement in quality of life metrics and reduction in HF hospitalization was present within the subsection of patients at prohibitive risk of MV surgery (49). An additional benefit of timely MV intervention was noted in the form of LV reverse remodeling and reduction in LV and left atrial volumes from successful reduction in MR using MitraClip according to the initial EVEREST II trial and subsequent data (50). Studies examining commercial use of MitraClip in predominantly *primary* MR have reported high procedural success with <3 grade residual MR of >90%, hospital mortality rate <3% and overall 30-day serious complication rates of 10 to 15% (51, 52). Based on these initial results, continued overwhelmingly favorable outcomes with MitraClip in *degenerative* MR over time and advancements in cardiovascular imaging enabling better anatomical characterization, an argument is made for wider clinical application of the device among patients at less than prohibitive risk of surgery. Such patient selection should occur on the basis of individualized decision-making and the Heart Team approach.

#### Chronic Secondary MR

**Figure 5:**
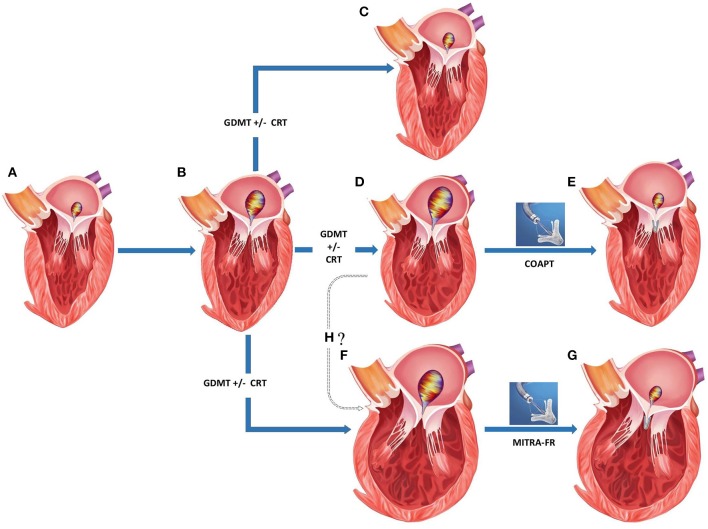
Appropriate patient selection for percutaneous transcatheter mitral valve replacement in severe functional mitral regurgitation (MR). **(A)** Normal left ventricular (LV) dimensions with mild MR. **(B)** Progression to moderate regurgitation and mild LV dysfunction. **(C)** Left ventricular recovery with mild MR following medical management. **(D)** Progression of LV dysfunction with mild-moderate LV dilation and severe MR despite medical management. **(E)** Post-MitraClip improvement in LV function, LV reverse remodeling, and reduction in residual MR. **(F)** Progression of LV dysfunction with moderate-severe LV dilation and severe MR despite medical management. **(G)** Post-MitraClip no change in LV size or function despite reduction in residual MR. **(H)** Whether structural changes in **(D,F)** represent separate phenotypes or a continuation on the spectrum of increasing disease severity. GDMT, guideline directed medical therapy; CRT, chronic resynchronization therapy.

Two separate randomized controlled trials compared the efficacy of percutaneous MV repair using MitraClip to medical therapy among patients with significant *secondary* MR and underlying LV abnormality [Table 5; (47, 48)]. These studies found conflicting results which can be explained to a certain degree by differences in their study design and patient enrolment (Figure 5).

##### Cardiovascular outcomes assessment of the mitraClip percutaneous therapy for heart failure patients with functional mitral regurgitation (COAPT Trial)

The study enrolled 614 patients with LV dysfunction (EF 20–50%) and moderate-to-severe or severe *secondary* MR who remained symptomatic despite the use of maximal doses of medical therapy. Among those enrolled in the trial, 302 patients were assigned to the MitraClip group (and guideline directed medical therapy) and 312 patients to the control group receiving just guideline directed medical therapy. The study excluded patients with LVESD >7 cm. Placement of MitraClip was successful in 98% of the treatment arm with 95% of the patients with echocardiograms at discharge showing <3 grade residual MR. Similarly at 12-months, severity of MR was <3 grade in 94.8% compared to 46.9% in the treatment and control arms, respectively. Clinical study endpoints were significantly improved in the treatment arm compared to controls, such as 2-year mortality (29.1 vs. 46.1%) and 2-year HF hospitalization (35.8 vs. 67.9% per patient year). Interestingly, there was no difference in mortality at 12-months between study groups. Additional measures of quality of life such as NYHA functional class I or II (72.2 vs. 49.6%) and change in the mean Kansas City Cardiomyopathy Questionnaire Score (+12.5 vs. −3.6 points) were more favorable among patients receiving MitraClip compared to the medical arm. There was evidence of reverse remodeling at 12-months within the treatment arm compared to control (mean change in LVEDV from baseline −3.7 ml vs. +17.1 ml, respectively). At 12-months, 3.4% of patients experienced a MitraClip related complication (composite of single leaflet attachment, device embolization, endocarditis or mitral stenosis needing surgery, left ventricular assist device implantation, cardiac transplantation, and device complication requiring non-elective cardiovascular surgery).

##### Percutaneous repair with the mitraClip device for severe functional/secondary mitral regurgitation (MITRA-FR trial)

The trial enrolled 304 symptomatic patients with LV dysfunction (EF 15–40%) and moderate to severe *secondary* MR to either percutaneous mitral-valve repair plus medical therapy (*n* = 152) or medical therapy alone (*n* = 152). Placement of MitraClip was successful in 95.8% of the treatment arm with 91.9% of the patients showing <3 grade residual MR at discharge and ≈82% at 12-months. Mortality (24.3 vs. 22.4%) and unplanned HF hospitalization (48.7 vs. 47.4%) at 12-months were similar in the device and control arms. Patient NYHA functional class I or II (range ≈65–70%) and mean EQ-5D quality of life score (60.8 vs. 58.6) ware similar between the treatment and control arms at 12-months. Improvement in NYHA functional class occurred in both arms compared to their baseline. Differences in median LVEDD, LVEDV, LVESD, and LVESV were minimal in both study groups at 12-months from baseline. Within the intervention group, 14.6% of patients experienced a MitraClip related complication (composite of device implantation failure, significant hemorrhage or vascular event, atrial septal lesion, cardiogenic shock requiring inotropes, cardiac embolism, tamponade, and urgent conversion to cardiac surgery).

### COAPT vs. MITRA-FR

Despite the overall similarities in trial design and high overall event rates signaling enrollment of a high risk patient population in both trials, there were several important differences that may have led to the disparate results (53, 54). One of the key variations was the criteria used for defining severity of MR. MITRA-FR used the 2014 ACC/AHA and ESC valvular guidelines where more modest degrees of MR were misclassified as severe *secondary* MR. We have represented several of the relevant baseline differences between device arms in the two trials as displayed in Table 6.

**Table 6:** Baseline characteristics in COAPT and MITRACLIP trials (device arms).

**Variable**	**COAPT MitraClip arm (*n* = 302)**	**MITRA-FR MitraClip arm (*N* = 152)**
Clip implantation success rate (implanted/attempted)	98% (287/293)	95.8% (138/144)
Inclusion criteria for degree of secondary MR^*^ Regurgitant volume Effective regurgitant orifice Grade of MR	>45 ml ≥0.3 cm^2^ ≥3+	>30 ml >0.2 cm^2^ ≥3+
Age – years (mean ± SD)	71.7 ± 10.1	70.1 ± 10.1
Male sex	66.6%	78.9%
NYHA class III/IV	57%	63.1%
Previous myocardial infarction	51.7%	49.3%
Previous atrial fibrillation	57.3%	34.5%
Type of Cardiomyopathy Ischemic Non-Ischemic	60.9% 39.1%	62.5% 37.5%
Medications at baseline ACEI, ARB or ARNI Beta-blocker Mineralocorticoid receptor antagonist Diuretic Oral anticoagulant	71.5% 91.1% 50.7% 89.4% 46.4%	83.0% 88.2% 56.6% 99.3% 61.2%
Previous cardiac resynchronization therapy	38.1%	30.5%
B-type natriuretic peptide level (pg/ml)	1,014 (Mean)	765 (Median)
≥2 clips implanted	61.8%	54.3 %
Effective regurgitant orifice area (cm^2^)	0.41 ± 0.15	0.31 ± 0.1
Mean left ventricular end-diastolic volume (ml)^¥^	194	254
Left ventricular ejection fraction (%)	31.3 ± 9.1	33.3 ± 6.5

* *Confirmed at an Echocardiographic Core Laboratory before enrollment corresponding to moderate-to-severe or severe MR*.

¥ *Calculated left ventricular end-diastolic volume in MITRA-FR arm based on indexed volume 136.2 ml/m^2^*.

*MR-mitral regurgitation, SD, standard deviation; ACEI, angiotensin-converting enzyme inhibitors; ARB, angiotensin II receptor blocker; ARNI, Angiotensin Receptor-Neprilysin Inhibitor*.

To summarize these differences, compared to patients in MITRA-FR, those in COAPT had

- More severe degrees of MR,
- Less remodeled LV,
- Disease refractory to medical therapy resulting in lower potential for improvement among controls,
- HF disease that could be attributed to valvular dysfunction over ventricular dysfunction,
- Improved procedural efficacy and less residual (grade <3) MR and
- Longer follow up in COAPT since differences emerged beyond the 12-month mark.

Additional trial information on guideline directed medical therapy, dose titration and CRT optimization would shed more light on the differences between the two trials. Confirmation of the clinical responsiveness to MitraClip implantation of proportionate vs. disproportionately severe MR could be confirmed by combining data from these trials and identifying response to specific disease phenotypes. More longitudinal data from MITRA-FR and publication of well-designed randomized control trials with distinct morphological entry criteria will pave the way for furthering our understanding on percutaneous MV repair in *secondary MR* and assist in exploring timing of such intervention. Whether coupling clip placement with other percutaneous procedures directed toward optimization of other anatomical abnormalities within the mitral apparatus (such as chordal replacement, altering ventricular, or atrial geometry among others) alters the disease course of severe MR remains to be studied and has potential to expand the patient pool that would benefit from such a combination procedure (55).

### Proportionate vs. Disproportionate MR

*Secondary MR* seems to represent a rather diverse group where in some situations, the MR can be explained by morphological changes affecting the LV such as global dilation and others when remodeling changes within the LV affect the MV apparatus to a greater degree than global LV function (56). In a recent paper, Grayburn and Packer described how EROA is dependent on both the LVEDV and LVEF, such that among patients with reduced LVEF ≈30% and LV dilation (LVEDV 200–250 ml), an EROA of 0.2 cm^2^ is common and reflects only a moderate degree of MR instead of severe (57). In such cases, MV intervention was unlikely to benefit as patients were considered to have a proportionate degree of MR to LV dilation. The authors raised a framework in which the severity of MR was identified by integrating the EROA, LVEDV, and LVEF, and responders to mitral valve intervention were felt to have an unexpectedly severe and disproportionate degree of MR to the degree of LV dilation. Responders to mitral valve intervention were more likely to have a less-dilated LV and relatively larger EROA, where the ratio of EROA to LVEDV was higher among patients with disproportionate MR than those with proportionate MR (Figure 5).

### Unresolved Questions

With two randomized trials of MitraClip in *functional* MR yielding differing results and despite attempts to reconcile the differences to pinpoint responders, there remain several unknowns.

- It is unclear if the phenotypical varieties of MR with disproportionately severe and proportionately severe MR represent disease on a spectrum of increasing severity and whether early intervention would prevent progression to the latter more advanced disease (Figure 5H). As an alternative theory, it is conceivable that these may exist as independent entities and in patients where cardiac remodeling disproportionately involves the muscle supporting MV, malcoaptation results in MR. Such MR is actually more responsive to MitraClip implantation and the result is an improvement in clinical outcomes with LV reverse remodeling.
- MitraClip placement and acute reductions in MR provides room for uptitrating well-validated heart failure medical therapies, and the gains from such medical optimization may play a role and will become apparent over time.
- Objective measurement of MR severity relies heavily on Proximal Isovelocity Surface Area Calculation (PISA) from flow convergence on 2-dimensional echocardiography. Existing data suggests that regurgitant jets in *functional* MR are often eccentric with asymmetrical flow convergence patterns inadequately visualized by the current standard in clinical practice i.e., 2-dimensional echocardiography. The EROA measurement may be more accurate using 3-dimensional imaging techniques with greater accuracy in recognition of the PISA radius (58). Direct cardiac imaging techniques such as computed tomography and magnetic resonance imaging have the ability to directly measure the EROA and regurgitant volumes (59). Whether severity parameters for MR can be used interchangeably across the newer modalities needs further testing prior to more widespread clinical use.
- Prior MitraClip trials did not enroll patients with significant tricuspid regurgitation and markedly elevated pulmonary arterial pressures. Innovations in percutaneous tricuspid valvular interventions may enable future interventions on both atrioventricular valves during the same or in a staged setting to maximize benefit (60).

As more prospective and retrospective analyses testing several of these previously mentioned hypotheses and theories come forward, our ability to understand and manage *functional* MR is destined to evolve.

#### Acute MR

There is limited experience with transcatheter MV repair in treatment of acute MR. Successful placement of MitraClip has been described in some patients developing severe MR following acute myocardial infarction with acute improvement in MR severity and symptoms (61). In one case series of 5 patients post-AMI, MitraClip was placed with marked symptomatic improvement and reduction in pulmonary pressures with <3+ residual MR in all patients. MitraClip was successfully placed with immediate reduction in MR severity and pulmonary pressures. One patient died of multi-organ failure within 1 week of the procedure. The other four patients were alive after 1 year with improved New York Heart Association (NYHA) functional class. While none of the patients in this case series had papillary muscle rupture accompanying acute severe MR, reports do exist on treating acute MR with MitraClip in the presence of posterior papillary muscle rupture (62). Widespread evidence for the benefit of percutaneous approach over surgical intervention and its longevity is still lacking and surgical intervention remains the most appropriate option in most patients with acute MR. Percutaneous intervention remains a tool in high surgical risk patients and requires assessment by a multidisciplinary team. Timing of such intervention remains unclear with some patients needing respiratory support in addition to short term mechanical circulatory support in the interim while awaiting decision and treatment.

### Emerging Technologies for Percutaneous Mitral Intervention

**Figure 6:**
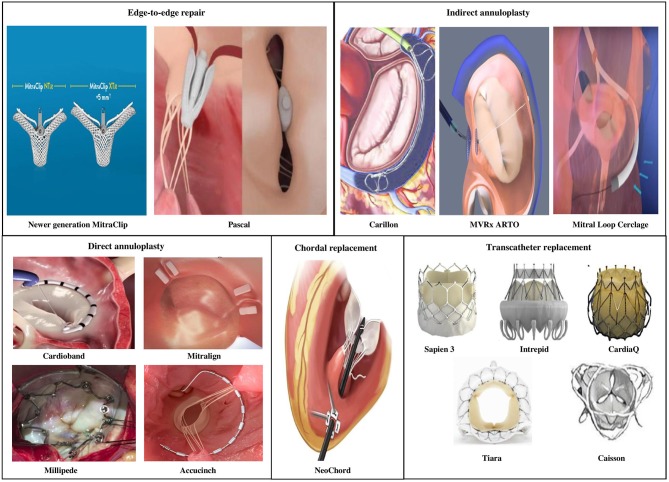
Newer transcatheter mitral valve interventions in patients with mitral regurgitation.

A considerable number of patients with severe MR do not meet anatomic criteria for MitraClip repair. Several other devices in the percutaneous arena possess potential applications in chronic MR permitting a customized strategy to mimic the traditional surgical interventions, in an individual or combination approach (Figure 6).

Currently there are four primary transcatheter approaches (Table 7)

- Edge-to-edge clip (Alfieri-type) repair (MitraClip, PASCAL TMVr system),
- Percutaneous MV annuloplasty indirectly via the coronary sinus or directly from retrograde LV access (Carillon, Cardioband, Millipede, Mitralign, ARTO systems),
- Chordal replacement (NeoChord, Harpoon Cords) and
- Transcatheter MV replacement (Sapien-XT, Melody, CardiaAQ, Caisson valve, etc.).

**Table 7:** Emerging transcatheter mitral valve repair technologies for mitral regurgitation with data in humans.

**Device**	**Access**	**Transseptal puncture**	**Etiology**	**Mechanism**
Edge-to-edge repair				
MitraClip PASCAL	Femoral vein Femoral vein	Yes Yes	PMR, FMR PMR, FMR	Clip based edge to edge repair. Creation of a double orifice mitral valve to reduce regurgitation
Indirect Annulopasty				
Carillon MVRx ARTO Mitral Loop Cerclage	Internal jugular Internal jugular and femoral vein Subclavian vein + femoral vein	No Yes No	FMR FMR FMR	Reduction in mitral regurgitation through reduction in mitral valve annulus through coronary sinus. Due to its proximity to left circumflax artery, coronary artery compression is a known complication
Direct Annulopasty				
Cardioband Mitralign Accucinch (Ventriculoplasty) Millipede	Femoral vein Femoral artery Femoral artery Femoral vein	Yes No No Yes	FMR FMR FMR FMR	Direct attachment to and reduction in mitral valve annulus to reduce mitral regurgitation. Placement may be within the left ventricular wall in case of certain devices
Chordal Replacement				
NeoChord	Transapical off-pump	No	PMR	Attachement of false chordae to mitral leaflets in cases of leaflet prolapse or flail to reduce mitral regurgitation
Transcatheter valve replacement				
Endovalve, Tiara, Fortis, Tendyne, etc. Sapien-XT	Transapical off-pump Femoral vein	No Yes	PMR, FMR Bioprosthetic valve dysfunction	Transcatheter bioprosthetic mitral valve placement either as valve in valve, valve in ring or valve in native mitral annulus to reduce mitral regurgitation
CardiaAQ, Caisson, Cardiovalve	Femoral vein +/– femoral artery	Yes	PMR, FMR	

*PMR, primary or degenerative mitral regurgitation; FMR, secondary or functional mitral regurgitation*.

Of these transcatheter techniques, only the former two have viable well-tested percutaneous access MV repair strategies. Most the upcoming technology on either chordal replacement or MV replacement uses primarily transapical access via lateral mini-thoracotomy.

### Edge-to-Edge Clip Repair

- **PASCAL Transcatheter MV Repair:** The PASCAL TMVr (Edwards Lifesciences, Irvine, CA) system is designed to overcome shortcomings of the MitraClip system by facilitating easy steering within the left atrium, larger implant size, broader paddles with central spacer within device to reduce MR by maximizing leaflet coaptation, ability to grasp individual leaflets and implant elongation to promote safe subvalvular maneuvering (63). Placement of the device occurs via transvenous access (femoral) and transseptal approach similar to the MitraClip. In its first human study, the PASCAL TMVr was studied in 23 patients with symptomatic severe *degenerative, functional* or *mixed* etiology MR (NYHA functional class III to IV) and patients were deemed high to inoperable surgical risk (64). Patients were not considered candidates for MitraClip repair either due to anatomical complexity (short posterior leaflet, large malcoaptation area, severe annular dilatation >61 mm) or lack of an approved indication for use. Procedural success was obtained in 22/23 patients (96%), residual MR was <3 grade in 96% patients and reduction in NYHA functional class ≤ II grade occurred in 95% of the cases. By 30-days post-implantation, three patients (13%) had died. Direct procedure related complications occurred in two cases (9%) from a minor bleeding event and transient ischemic attack, respectively. The device expanded patient eligibility for repair especially in case of short posterior leaflets and larger flail gaps and needs additional data on durability and future head-to-head comparisons with newer generations of the MitraClip device.

### Indirect Annuloplasty Devices

- **CARILLON Mitral Contour System:** The CARILLON Mitral Contour System (Cardiac Dimensions, Inc., Kirkland, Washington) combines a proprietary, implantable device with a percutaneous catheter delivery system through transjugular venous access to treat *functional* MR. The device is an indirect annuloplasty device composed of two self-expanding nitinol anchors with a connecting curvilinear segment and is positioned with its proximal anchor at the coronary sinus ostium, distal anchor within the great cardiac vein. Upon deployment the device plicates the tissue next to the MV annulus reducing mitral annular dilation and degree of MR by bringing the anterior and posterior leaflets closer. Coronary angiography is also performed to evaluate for left circumflex-obtuse marginal arterial system compression following deployment due to its close proximity. Current technology allows either recapture and repositioning of device implant during same procedure or recapture and removal of original device followed by new device implantation when needed. Such manipulation becomes possible due to its benign design and availability in multiple sizes.
- Initial studies of the CARILLON Mitral Contour System, AMADEUS, and TITAN showed improvement in symptoms, quality of life, severity of MR and evidence for LV reverse remodeling when used to treat symptomatic high risk patients with FMR (65, 66). The earlier generation device had a reasonable safety profile, but asymptomatic wire-form fractures were seen at the level of the high strain proximal anchor locking mechanism in the Carillon device in 25% of cases. TITAN II was a prospective, single-arm, multinational safety study conducted in order to test the newer generation modified Carillon device designed to reduce the strain in the wireforms of the proximal anchoring segment (67). There was a single device fracture 1/36 (2.8%) attributed to incorrect placement of a recaptured/redeployed device. The primary end point of 30-day major adverse event rate was 2.8% due to one incidence of non-arrhythmic sudden death occurring at 17-days post-procedure. The 1-year mortality was 23% (7 of 30 patients) and no deaths were adjudicated to be device related. From the efficacy standpoint, TITAN II showed similar clinical and echocardiographic benefits as in TITAN with reduction in MR, mitral annular dimension, improvement in NYHA functional class and a trend toward reduction in ventricular size suggestive of reverse remodeling. The modified Carillon device used in the TITAN II study is currently being evaluated in a multicenter blinded randomized control trial (REDUCE FMR trial).
- **ARTO device: The MVRx ARTO transcatheter annular reduction therapy** (MVRX, Inc., Belmont, California) is an indirect annuloplasty system that includes transvenous delivery of 2 anchors: one through the interatrial septum, the other to the coronary sinus and acts by reducing the anteroposterior diameter of the mitral annulus. Procedure is performed using general anesthesia. The MV RepaIr Clinical (MAVERIC) trial program is a prospective single arm group of studies evaluating safety and performance of the device in *functional* MR with promising early results in the first 11 of a total of 31 patients (68). Publication of full data is awaited.
- **Mitral Loop Cerclage Catheter System:** In the mitral loop cerclage (Tau-PNU Medical Co, Ltd., Pusan, Korea) procedure, both the femoral vein and left subclavian vein (via a pacemaker-type pocket) are accessed. The cerclage is accomplished by using a guidewire to enter the coronary sinus and great cardiac vein, crossing the interventricular septum from the anterior interventricular vein into the right ventricle, snaring this wire from the right ventricular outflow tract and forming a loop around mitral annular plane. The guidewire is exchanged for a tension device containing an integrated coronary artery protection element preventing coronary compression and tension is applied to compress the mitral annulus and improve leaflet coaptation. The tension locking device is embedded in the left subclavicular pocket. The procedure is performed either under general anesthesia or moderate sedation. The first in human study attempted the procedure in 5 patients, was successful in 4/5 patients but aborted in one due to unfavorable anatomy (69). The device resulted in immediate reduction in MR that was sustained upto 6-months and reduced left atrial and LV chamber volumes over time. Device related complications were coronary artery occlusion, new bundle branch block and need for a repositioning procedure. While several breakthroughs are being made in the field of indirect annuloplasty to reduce *functional* MR, unfavorable coronary sinus and branch vein anatomy seems to play a major role in limiting procedural feasibility in a significant proportion of patients depending on device.

### Direct Annulopasty Devices

- **Cardioband:** The Cardioband device (Edwards Lifesciences, Irvine, CA) delivers direct sutureless anchors around the mitral annulus to connect the annuloplasty device. The cardioband system enables adjustable septo-lateral diameter compression, reducing MV annulus size and severity of MR. In the largest multicenter study of 60 patients with moderate to severe *secondary* MR who underwent Cardioband implantation, early results raised issue with device design leading to device modification half way through the study (70). Anchor disengagement was observed in 10 patients, resulting in device inefficacy in five patients but most (9/10 anchor disengagement) occurred prior to device modification. There were no device related deaths and 1-year overall survival was 87%. While severity of MR improved in most patients at 1-year, worsening of MR was still noted in 1/5 patients. Quality of life markers, exercise capacity and NYHA functional status improved at 1-year compared to baseline in most patients. The 2-year (unpublished) results continue to reveal sustained reduction in septolateral diameter, MR severity and patient quality of life.
- **Mitralign Annuloplasty system:** The Mitralign system (Mitralign, Tewksbury,Massachusetts) involves transfemoral access using a deflectable catheter which is introduced into the LV and directed toward the posterior annulus. Using a combination of wires and catheters, polyester pledgets are placed across the annulus into the left atrium first at the P1/P2 scallops followed by the P2/P3 scallops of posterior mitral annulus if needed. One to two pairs of pledgets are plicated, locked and the result is a reduction in MV annular diameter. The device has been tested in a prospective, multicenter single-arm feasibility study where 45 patients underwent procedure (71). There were no intraprocedural deaths or conversion to surgery, but pericardial tamponade occurred in 4 (8%) patients. Exclusion of LVEDD <5 cm and second generation catheter systems have decreased the risk of tamponade. Within 6-months, all-cause mortality was 12.2%, 7 (17%) patients underwent MitraClip placement and one patients received non-emergent MV surgery. Improvement was noted in MR severity in 50% of the patients with worsening in 15.4% cases, with greater trend for improvement in those who received 2 pledgets. HF symptoms and 6-min walk test improved at 6-months from baseline. It is not currently available for commercial use.
- **Newer devices**: The Millipede IRIS ring is a semirigid “zigzag” shaped annuloplasty ring, with eight helical stainless steel anchors that anchor directly into the mitral annulus. Device has eight tensioning sliders that can be used to actuate the device and reduce the annulus size (72). The AccuCinch Ventricular Repair System (Ancora Heart, Santa Clara, CA) uses a retrograde arterial mechanism to implant a series of adjustable anchors within the LV wall tethered by a cable below the mitral valve annulus. The cable is tightened to cinch the left ventricular wall, reducing ventricular size and consequently mitral annulus, thus succeeding in lowering regurgitant volume. Unlike other systems within this section, the AccuCinch system represents more of a ventriculoplasty than direct annuloplasty due to its direct support to and placement within the left ventricular myocardium; consequently, this device is current being tested in heart failure patients with dilated left ventricles but without significant valvular lesions. Prospective clinical data is awaited on these devices and studies are underway.

### Chordal Replacement

The Neochord is a transcatheter surgical off-pump mitral repair procedure which implants artificial cords into the mitral valve and is performed under general anesthesia in a standard cardiac operating theater. Access to the LV is obtained through a left lateral mini-thoracotomy and transapical access. Several studies on the safety and efficacy of such a transcatheter strategy in reducing MR have been published (73, 74). Another similar device is the Harpoon MV Repair System that anchors artificial cords on the flaps to take the place of the natural cords via transapical off-pump surgical technique using transcatheter technology. Chordal replacement is more commonly used in *degenerative* MV disease and no current transvenous or transarterial systems mimic either of these techniques.

### Transcatheter MV Replacement

There have been several studies demonstrating feasibility of transcatheter MV replacement using a bioprosthetic valve for symptomatic MR especially among high risk surgical patients (75, 76). These new transcatheter valves are mostly implanted via minimally invasive surgical approach and transapical access but recent literature sheds light on promising new percutaneous transseptal delivery systems (77). We have directed our focus below primarily to the percutaneous non-surgical implantable valvular systems. As more feasibility data becomes available, these results show favorable reduction in MR severity and improvement in patient symptoms with an acceptable early mortality rate among high surgical risk populations. Challenges remain due to complex mitral anatomy, proximity to LV outflow tract, valve positioning, mitral annular calcification, large delivery systems and valve size, device thrombosis, and hemolysis in addition to complications from transapical access.

Complementing the significant strides achieved in aortic valve implantation, techniques required to perform mitral transcatheter implantation have progressed quickly. The SAPIEN-XT (Edwards Lifesciences, Irvine, CA) and Melody valve (Medtronic, Minneapolis, MN) have shown excellent success in percutaneous valve-in-valve and valve-in-ring implantations in the mitral position but are not recommended in native regurgitant MVs (78, 79). Placement of bioprosthetic MV through vascular access has been challenging primarily due to the larger device size in the mitral position, an asymmetric D-shaped dynamic annulus and lack of adequate support from the native MV annulus. While clinical data is scant, percutaneous mitral replacement into native valves has been successful in certain cases. The CardiaAQ (CardiAQ Valve Technologies, Inc. Winchester, MA) valve has leaflets made from porcine pericardium onto a nitinol self-expanding stent and was delivered transseptally in an 86-year old high risk patient with improvement in MR (80). Four separate vascular accesses were obtained, 2 in the femoral artery and 2 in the femoral vein to facilitate the complex procedure using multiple catheters and delivery systems. The patient later died on day 3 post-procedure from non-device related complications. In a first of its kind study, PRELUDE studied feasibility of transfemoral access and transseptal delivery of the Caisson transcatheter MV replacement (LivaNova, Maplegrove, MN) in humans. While the results have not been published, statements released from the company indicated encouraging positive outcomes with sustained valvular performance and improved quality of life in patients post-replacement. The INTERLUDE CE-Mark clinical trial has been launched using this device to be performed at sites across North American and Europe. In a first in human study, 10 patients underwent percutaneous transcatheter mitral valve replacement via transseptal approach for severe MR of varying etiology (4 *degenerative*, 4 *functional*, 2 *mixed*) and high surgical risk (77). The delivery system comprises a nitinol dock encircling the chordae tendineae, and a balloon-expandable bioprosthetic valve. The device was successfully implanted in 9 of the 10 patients. Residual MR was mostly trivial ( ≤ 1+ MR) in all nine patients that underwent valve replacement with a minimal transmitral gradient. At 30 days, there was no death, stroke, myocardial infarction, re-hospitalization, left ventricular outflow tract obstruction, device migration, embolization, or conversion to mitral surgery. Complications reported were a case of pericardial effusion precluding valve placement and one case of paravalvular regurgitation managed with a percutaneous closure device.

As several companies are developing percutaneous systems for delivering bioprosthetic MVs, trends indicate that the coming 5-years will see rapid advancements in the field of percutaneous MV replacement and more human data will become available from ongoing studies (81). Within the next decade, it is certainly plausible that we will see studies among low to intermediate surgical risk populations as transcatheter techniques evolve and achieve greater success.

## Conclusion

Heart Team sets the foundation for delivering the best quality of care to patients with valvular heart disease by leveraging the expertise of its members and enhancing collaboration. Technical and more so technological advances have forged the field of microinvasive cardiac valvular operation and amplified the role of a Heart Team approach (82, 83). These procedures include a variety of percutaneous or transapical transcatheter valve repair and replacement systems that can be implanted without cardiopulmonary bypass often requiring only local anesthesia. Percutaneous transcatheter valve repair has especially become increasingly feasible, with a remarkable safety profile and a broadening clinical applications. While surgery remains the treatment of choice in *degenerative* MR, COAPT, and MITRA-FR have greatly enhanced our knowledge on intervention in *functional* MR. These data come as a relief after years of no clear direction timing of mitral valvular intervention and the role for percutaneous repair in patients with chronic severe *functional* MR. We also await results from the currently enrolling RESHAPE-HF2 randomized controlled trial to confirm or reject previously mentioned hypotheses on response to MitraClip treatment in severe functional MR. Results from COAPT will especially set the benchmark for future trials in the field of percutaneous mitral repair. Beyond the MitraClip, data comes from smaller experiences and there essentially is a crowding of percutaneous devices waiting to set themselves apart as more large-scale clinical trial data comes to light. We have embraced these new technologies and continue to witness expansion in minimally invasive transcatheter techniques with better safety and efficacy profiles over time that challenge current standards and greatly assist in caring for patients across several spectrums for surgical risk. Several of the MV percutaneous MV repair methods complement each other and may have longer-term durability and greater clinical impact. Evolutions in imaging technologies and fusion of 2D/3D echocardiographic with fluoroscopic imaging, allowing simultaneous viewing and superimposition of the different techniques, will further enhance safety, lower complication rates, shorten procedure times, accelerate achievement of technical expertise and optimize execution of these minimally invasive percutaneous procedures.

## Author Contributions

MS and UJ were involved in the planning, the writing of the manuscript, and making the figures and the tables.

### Conflict of Interest Statement

The authors declare that the research was conducted in the absence of any commercial or financial relationships that could be construed as a potential conflict of interest.
